# Sustainable Preparation of Graphene Quantum Dots for Metal Ion Sensing Application

**DOI:** 10.3390/nano13010148

**Published:** 2022-12-28

**Authors:** Asif Saud, Haleema Saleem, Nazmin Munira, Arqam Azad Shahab, Hammadur Rahman Siddiqui, Syed Javaid Zaidi

**Affiliations:** UNESCO Chair on Desalination and Water Treatment, Center for Advanced Materials, Qatar University, Doha P.O. Box 2713, Qatar

**Keywords:** graphene quantum dots, eucalyptus trees, hydrothermal treatment, leaf extracts, photoluminescence, metal sensing

## Abstract

**Highlights:**

**What are the main finding?**

- Preparation of GQDs from ethanolic extract of eucalyptus tree leaves.
- Hydrothermal treatment of extract at different temperatures and times was carried out.

**What is the implication of the main finding?**

- The GQDs were formed in the size range of 2–5 nm, as validated by TEM images.
- Developed GQDs were successfully used for metal ion sensing.

**Abstract:**

Over the past several years, graphene quantum dots (GQDs) have been extensively studied in water treatment and sensing applications because of their exceptional structure-related properties, intrinsic inert carbon property, eco-friendly nature, etc. This work reported on the preparation of GQDs from the ethanolic extracts of eucalyptus tree leaves by a hydrothermal treatment technique. Different heat treatment times and temperatures were used during the hydrothermal treatment technique. The optical, morphological, and compositional analyses of the green-synthesized GQDs were carried out. It can be noted that the product yield of GQDs showed the maximum yield at a reaction temperature of 300 °C. Further, it was noted that at a treatment period of 480 min, the greatest product yield of about 44.34% was attained. The quantum yields of prepared GQDs obtained after 480 min of treatment at 300 °C (named as GQD/300) were noted to be 0.069. Moreover, the D/G ratio of GQD/300 was noted to be 0.532 and this suggested that the GQD/300 developed has a nano-crystalline graphite structure. The TEM images demonstrated the development of GQD/300 with sizes between 2.0 to 5.0 nm. Furthermore, it was noted that the GQD/300 can detect Fe^3+^ in a very selective manner, and hence the developed GQD/300 was successfully used for the metal ion sensing application.

## 1. Introduction

With the world’s rapid development in several sectors, the possibility of excessive pollution ejection into the environment has increased [1,2,3]. As a result, environmental water pollution caused by harmful metal ions has developed as one of the most serious issues faced by humans [4,5]. In every aspect of daily life, water is crucial and as a result, it is extremely important to have access to clean and potable water. Wastewater such as produced water can be treated properly for various applications. Polluted water might contain hazardous metal ions such as Pb^2+^, Hg^2+^, Fe^3+^, Fe^2+^, Cu^2+^, Cd^2+^, Co^2+^, Ni^2+^, Al^3+^, and Ag^+^ [6,7,8,9,10], and these metal ions can combine to produce dangerous solution compounds which can turn out to be toxic [11,12,13]. These toxic compounds do not disintegrate and are non-degradable. As a result, these compounds keep building up in the soil and eventually reach human bodies [14,15]. Since these problems have become a serious issue, the field of toxic metal ion sensing has become more popular among the research communities [16,17,18]. To detect harmful metal ions, numerous types of sensing systems combined with various materials have been developed, and graphene-based materials are having good potential in sensing applications [19,20,21,22]. The main limitations of the conventional metal sensing methods include low selectivity, instability, difficult on-site sampling, and poor compatibility in the aqueous environment. Given that metal ions have a direct relationship with environmental water pollution, it is extremely desirable to design and develop effective as well as selective fluorescent sensors for metal ion detection applications [22]. Fe^3+^ ions have always drawn a lot of attention among the different heavy metal ions, as it is difficult to identify and remove them. In general, it is necessary to monitor and maintain a balanced iron proportion in biological as well as environmental systems [23]. As metal ions might cause adverse impacts on human as well as environmental health, the usage of GQDs as fluorescent probes to detect metal ions has received good research attention recently. The metal ion Fe^3+^ performs an important role in both biological as well as ecological processes. In biological systems, Fe^3+^ ions might coordinate with several types of regulatory proteins. Excessive Fe^3+^ ions may lead to cytotoxicity, creating their concentration a vital marker for Parkinson’s disease. Therefore, the proper monitoring of Fe^3+^ pollution is very crucial [24]. Hence, there is a requirement for highly sensitive and specific sensors of Fe^3+^ concentrations in both the environment and biological systems. Due to its fascinating characteristics, such as chemical inertness, low toxicity, high solubility, persistent photoluminescence (PL), good surface grafting, etc., graphene quantum dots (GQDs) have recently begun to be recognized as a material to develop an advanced novel type of fluorescence sensor. There are several studies carried out using GQDs for improving water treatment efficiency and works related to the environmental impact of nanomaterials [25,26]. 

GQD is a zero-dimensional (0-D) member in the carbon-based nanomaterials family and is generally considered a shredded fragment from a graphene sheet. This material has been studied widely since its unforeseen discovery in 2004, during the carbon nanotube’s purification, and it has a honeycomb structure with a single carbon layer [27]. It has exceptional electrical, structural, chemical, and tunable optical properties of PL as well as electrochemiluminescence. The better stability, biocompatibility, superior dispersibility, surface grafting, good solubility, non-toxicity, and inertness of the GQD materials enable its extensive potential in several applications [28]. In recent times, GQDs have been utilized to identify several analytes based on their luminescence characteristics. Many sensors have been based on the GQDs’ photoluminescence quenching process [29,30]. The application of optical characteristics of GQDs for dangerous metal ion sensing has gained the attention of researchers since they are very compatible in aquatic environments, inexpensive, straightforward, quick, efficient, and highly sensitive and selective [31,32,33]. Numerous studies on the integration of GQDs with the aforementioned optical methods have been completed successfully to efficiently identify harmful metal ions. These studies were motivated by the beneficial optical properties of GQDs [34,35,36,37,38,39]. Different preparation methodology has been utilized in the preparation of GQDs, and it can be categorized into two types, respectively, (1) top down and (2) bottom-up methods [40]. The top-down method involves the breaking down of bulk materials into small-sized nanostructured materials, and it included methods such as chemical exfoliation [41], solvothermal preparation [42], nanolithography [43], and electrochemical cutting [44]. Contrarily, the bottom-up method involving in the formation of bigger units from small units, and it comprises techniques such as the cage-opening technique [45], hydrothermal heating [46], microwave irradiation [47], or thermal combustion [48]. In spite of the fact that the above-mentioned techniques have several benefits, these preparation methods require complex purification techniques, harmful organic solvents, low quantum yield, elevated temperature, treatment with concentrated acid or alkali, and superior quality carbon precursors. Even though different naturally occurring carbon species such as carbon black [49], carbon fibers [50], coal [51], etc., can be used as a precursor for the preparation of GQDs, these materials are also associated with fossil fuels which are considered as a non-renewable source and may have the possibility that it will not be adequately available in future. Therefore, green chemistry methods are used recently for the preparation of nanomaterials, which contribute additional benefits such as the possibility of large-scale production, biocompatibility, unique morphologies, environmental-friendly preparation, economic nature, the vast availability of different carbon sources, and the possibility of recycling the waste products into beneficial products [52]. 

The green-synthesized GQDs from various carbon sources such as mango leaves [53], tea waste [54], flower extract [55], cow’s milk [56], etc., were already reported. The utilization of the above-mentioned precursors for the preparation of GQDs has several benefits such as non-toxicity, easy handling nature, and large-scale availability. The results from the above-mentioned studies encouraged us to investigate whether we can develop the GQDs from green plants, which are the foundation of the majority of the Earth’s ecologies. Certain biomolecules, proteins, polysaccharides, vitamins, and enzymes in plants have a good capability for performing reduction as well as capping of non-biocompatible materials. Plant-based materials are proven to be an exceptional source for the bio-based preparation of carbon nanomaterials since these materials have high carbon content for the preparation of carbon-based nanomaterials. In recent times, carbon nanotubes have been synthesized from natural sources such as turpentine and eucalyptus [57]. Eucalyptus trees are now grown all over the globe and used extensively for their medicinal properties. Eucalyptus tree leaves are a byproduct of the trees, and these byproduct leaves can be used for nanomaterial synthesis and hence turn out to be a significant research subject. The leaf extracts of the Eucalyptus tree are mostly comprised of hydrocarbons having low oxygen content [58], and this will enable the leaf extract to be an ideal precursor to synthesize the GQDs. Dubeey et al. [59] claimed the application of methanol extract of eucalyptus hybrid leaves for the extra-cellular green preparation of silver(Ag) nanoparticles. In research carried out by Weng et al. [60], hybrid nonoxidized graphene oxide or iron-based nanoparticles were prepared by means of a green synthesis technique, using a single-step process with the leaf extract of Eucalyptus trees. Moreover, Zhuang et al. [61] prepared reduced graphene oxide from Eucalyptus leaf extract as these leaves are abundant in nature and the oxidized products are eco-friendly. According to another research by Wang et al. [62], the group illustrated that the aqueous leaf extracts of Eucalyptus trees were employed as a stabilizing and non-oxidizing agent for the preparation of iron nanoparticles.

The above-mentioned studies encouraged us to select the eucalyptus tree leaves as the precursor for the green preparation of GQDs. To the best of our knowledge, the leaf extract of Eucalyptus trees has never been used as the precursor for the sustainable development of GQDs to be used in metal ion sensing applications. This is a very sustainable way of preparing the GQDs, where the ethanol used will be recovered during the GQD preparation stage itself. The formation of GQDs was confirmed by UV–Visible spectrophotometry and PL. The morphology, as well as the surface properties of green-synthesized GQDs, were characterized by different techniques such as transmission electron microscopy (TEM), Fourier transform infrared spectroscopy (FTIR), X-ray diffraction analysis (XRD), and Raman spectroscopy analysis. The major objectives of the present work were to: (1) prepare the GQDs from eucalyptus leaves (2) characterize the green synthesized GQDs using different techniques and (3) use the developed GQDs for metal sensing application.

## 2. Experimental Section

### 2.1. Materials

Eucalyptus tree leaves were acquired from the Eucalyptus tree on the university grounds of Qatar University, Qatar. The ethanol was procured from Sigma Aldrich, St. Louis, MO, USA. Distilled water and ethanol used were obtained from Merck, Kenilworth, NJ, USA. The salts AgCl, FeCl_3_, NaCl, LiCl, AlCl_3_, CaCO_3_, CuSO_4_, CoCl_2_, CrCl_3_, MgSO_4_, SrCl_2_, MnCl_2_, MoCl_2_, FeCl_2_, ZnCl_2_ were supplied by Sigma Aldrich, St. Louis, MO, USA. Quinine sulfate and sulfuric acid were purchased locally from Sulfur chemicals, Doha, Qatar.

### 2.2. GQD Synthesis from Leaves of Eucalyptus Tree Employing Ethanol

**Figure 1:**
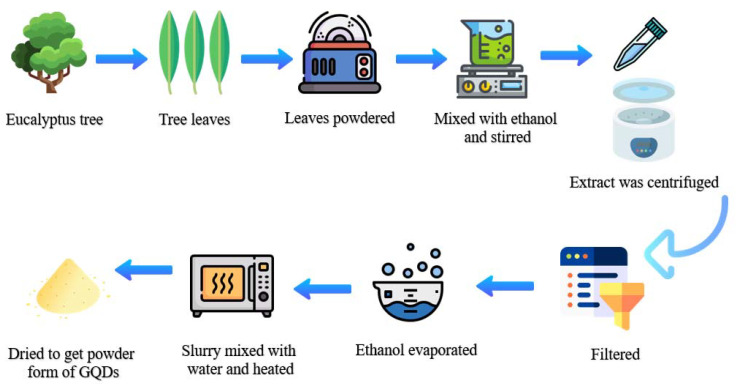
Graphical diagram for the preparation of GQDs from Eucalyptus tree leaves.

Eucalyptus tree leaves were collected, dried in oven for 5 h, and then ball milled the leaves to obtain fine powder. Subsequently, 10 gms of Eucalyptus tree leaves powder was mixed with pure ethanol solution, and this mixture was placed for continuous stirring for a time of 4 h at room temperature, and the resulting extract was centrifuged for 10 min at 8000 rpm for achieving a fine supernatant. Further, the obtained extract was filtered using a 0.22 micrometer filter and consequently concentrated by the evaporation of ethanol using a rotary evaporator till the residue slurry was obtained. This slurry was blended with a less sum of milli-Q water and heated in an oven at different temperatures (240, 260, 300, 320 °C) for different treatment times (360, 400, 440, 480, and 520 min), and subsequently, all sample residue was dispersed in absolute ethanol for properly dispersing the GQDs. The obtained dispersion was then filtered out using the syringe filter (0.22 μm) for obtaining the pure GQDs. These GQDs were then permitted for drying for 24 h at 65 °C to obtain the dried powder. The graphical diagram for the GQD synthesis from Eucalyptus tree leaves is shown in Figure 1.

### 2.3. Characterization of the Prepared GQDs

The FTIR instrument used in the current study was 760 Nicolet, and it permitted the identification of inorganic and organic groups present in the sample, based on their particular IR frequency. The X-ray diffraction pattern of the developed GQDs was obtained using XRD: PANalytical, EMPYREAN using Cu/Ka radiation with a 1.54-angstrom wavelength. To examine the degree of structural defects and crystallite size of GQDs, Raman analysis was carried out, with a laser power of 10 mW and wavelength of 532 nm. A fluorescence spectrophotometer was used to investigate the photo-luminescence behavior of GQDs. (Horiba FluoroMax-4 Spectrofluorometer), and absorption spectra were recorded in a UV−Visible spectrophotometer (Biochrom). The GQD morphology was examined employing TEM (HT 770, Hitachi, Japan).

### 2.4. Product Yield and Quantum Yield of the Prepared GQDs

The product yield of the developed GQDs was determined using Equation (1), below.

(1) $$Yield\left(\%\right)=\frac{Weight\;of\;Dried\;GQD\;obtained}{Weight\;of\;Slurry\;}\times 100$$

The quantum yield (QY) of the developed GQDs was determined using Equation (2), below.

(2) $$\Phi =\Phi_{r}\frac{I\left(Ar\right)n^{2}}{I_{r}\left(A\right)n_{r}^{2}}$$

where Φr and Φ are the QYs of the standard reference and sample, I_r_ and I are the integrated photoluminescence intensities of the reference and sample, A and A_r_ are the absorbance values of sample and reference, and n and n_r_ are the refractive indices of the sample and reference, respectively.

### 2.5. Application of GQD/300 as PL Metal Ion Sensor

In the present research work, the selective sensing of Fe^3+^ was carried out by 55 μg mL^−1^ GQD/300 solution employing 100 µM concentration of distinct metal ions: 100 µM concentrations of the following metal ions to accomplish the selective sensing of Fe^3+^: Co^2+^, Zn^2+^, Ag^+^, Na^+^, Mg^2+^, Mo^2+^, Sr^2+^, Fe^3+^, Li^+^, Ca^2+^, Mn^2+^, Al^3+^, Cr^3+^, Fe^2+^.

## 3. Results and Discussion

In the following section, we discuss the results of the optical, morphological, and structural characterization of GQDs, developed from the Eucalyptus tree leaves. Moreover, the product yield and quantum yield of the developed GQDs were also determined. Additionally, the efficiency of the developed GQDs in the metal sensing application. 

### 3.1. Optical Characterization of GQDs

**Figure 2:**
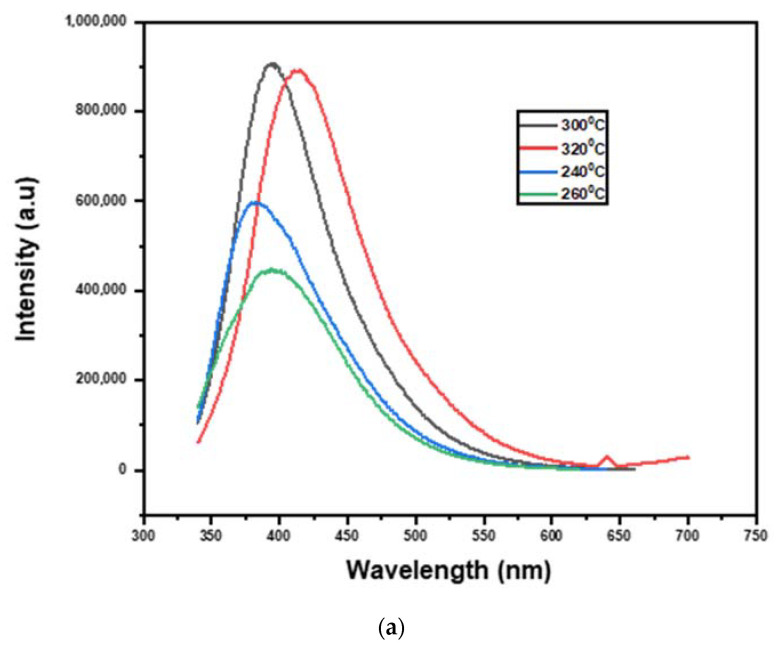

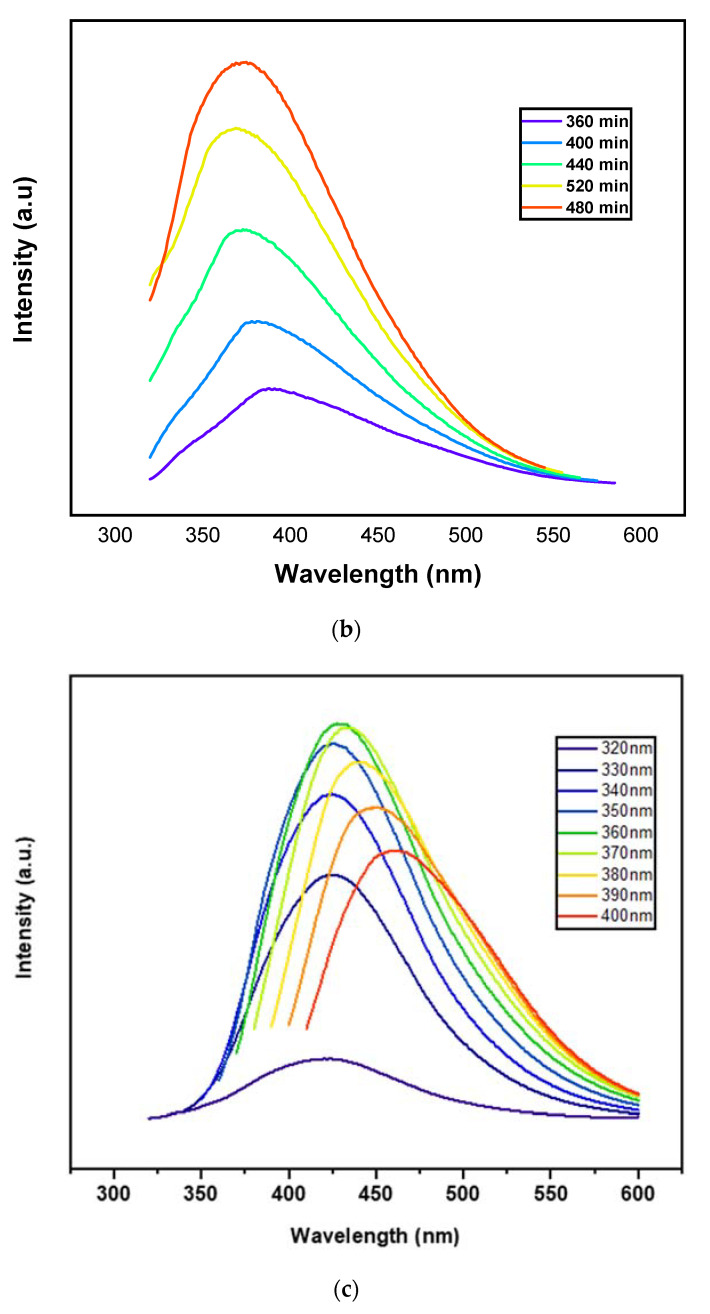
(**a**) PL of GQD at different temperatures (240, 260, 300, and 320 °C), (**b**) PL of GQD at different heat-treatment durations (360, 400, 440, 480, and 520 min) at a constant temperature of 300 °C (**c**) PL of GQD prepared at 300 °C after 480 min of treatment.

In the current study, a detailed study was performed at various temperatures for determining the effective work parameters (240–320 °C) and processing times (360 min) by observing the products’ PL emission and optical characteristics. To compare the PL intensities of the GQDs produced at various temperatures, same concentration of 55 g mL^−1^ was adopted. The PL emission intensities of the developed GQD at various temperatures (240, 260, 300, and 320 °C) at a predetermined period of time of 120 min was investigated and presented in Figure 2a. It was noted that the products obtained at 260 °C temperature showed less intense optical emission, indicating that below this temperature the cutting of carbon domains to develop GQDs will not induce good results. Therefore, the temperature was further increased to 320 °C [63,64]. It was observed in Figure 2a that the PL emission intensity enhanced with temperature up to 300 °C, whereas it declined with an additional increase in temperature to 320 °C. Additionally, the PL emission intensities of the developed GQD at different heat-treatment durations (360, 400, 440, 480, and 520 min) at a constant temperature of 300 °C are shown in Figure 2b. It was noted that the PL intensity enhanced with an increment in the treatment time up to 480 min and reduced thereafter (Figure 2b). These results suggest that increasing the temperature and the processing time can speed up the transformation of carbon domains into nanosized GQDs and enhance optical emission. Exceeding a certain temperature and processing time cutoff point might lead to GQD’s surface degradation and structural degradation and reduced PL emission. According to these results, it can be confirmed that the optimal conditions for the preparation of good quality GQDs with superior optical properties are a temperature value of 300 °C and a processing time of 480 min. 

The PL spectra of the developed GQD/300 are shown in Figure 2c. Examining the photoluminescence spectra of the generated nanostructured material at various excitation wavelengths between 320 and 400 nm revealed that the emission intensity increased to 360 nm and then decreased. The photoluminescence emission intensity decreased in direct proportion to the increase in excitation wavelength. Since the excitation wavelength increases from 300 to 400 nm, the photoluminescence peaks change to larger wavelengths, indicating a redshift (430 nm to 480 nm) [65]. At 360 nm wavelength, the excitation-dependent photoluminescence of GQDs has been observed. The highest PL intensity of GQD/300 is 434 nm, with a vibrational relaxation or dissipation of the wavelength at 174 nm. All of the GQD fluorescence studies are consistent with what has been reported in the previous findings [24,66]. The presence of conjugated aromatic hydrocarbons, existence of hydroxyl and other functional groups containing oxygen, emission of inhibited zigzag edge with carbine-like triple ground state as well as the emission that is trapped on the surfaces are considered to be the major reasons for the fluorescence emission mechanism of GQDs [67,68,69]. The PL characteristic of GQDs at excited state might be due to optical selection of GQDs at different sizes and defects of GQDs on the surface level [70,71]. According to a study [72], the key reason for the fluorescence in excited stage is that when the emission occurs from the carbon backbone of sp^2^, the sp^2^ conjugated domain of GQDs is adequate to have a limited energy gap in the band due to the effect quantum confinement.

**Figure 3:**
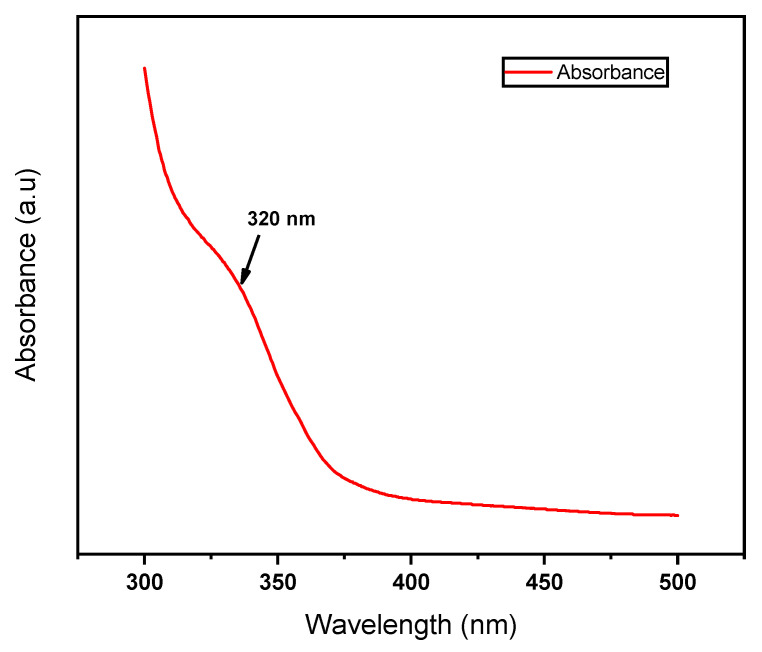
UV–Vis of GQD/300.

To additionally understand, the optical properties of GQDs prepared at 300 °C after 480 min of treatment (named GQD/300), these developed GQDs were examined thoroughly by UV–Vis absorption as well as photoluminescence emission spectroscopies. The optical properties of synthesized GQD/300 were explored by conducting the UV–Vis absorption. Usually, the absorption spectrum of graphene quantum dots appears in the UV region, and the tail extends toward the visible region. UV–Visible spectrum of GQD/300 as in Figure 3 shows a significant absorption at 300–320 nm which can be related to the π-π * and n-π * transition arising from aromatic C=C bonds (sp^2^ domain) and C=O groups, respectively [73]. The results suggested that the UV absorbance in the GQDs is associated with their surface oxygenated (C=O) states established at the time of reaction [74]. 

### 3.2. Product Yield of GQDs

A thorough investigation was carried out to examine the impact of temperature and reaction time on the GQD product yield. In accordance with the precursor of the eucalyptus extract, the product yield for each treatment was determined, and the results are summarized in Table 1. It can be noted that the product yield of GQDs showed the maximum yield at a reaction temperature of 300 °C. This GQD of reaction temperature of 300 °C was checked at different reaction times (Table 2), and it was noted that at a treatment period of 480 min, the greatest product yield of about 44.34% is attained. 

According to these findings, greater temperatures may cause carbon domains to cut into GQDs more quickly. These are the greatest yield figures for products that have been reported thus far for the preparation of GQDs from biomass waste to the best of the authors’ knowledge [75,76,77]. The yield of GQDs made from the various biomass-based precursors covered in the prior studies is summarized in the table.

### 3.3. Quantum Yield of GQDs

Quantum yield is considered to be a significant aspect related to the PL of GQDs. In most cases, the QY depends on the synthesis techniques as well as surface chemistry. By comparing the integrated PL intensities and absorbance values of GQDs and quinine sulfate, the quantum yields of GQDs were estimated. A quantum yield of 0.53 was shown by dissolved quinine sulfate in 0.1 M H_2_SO_4_. To prevent re-absorption effects, as-prepared GQD/300 was dissolved in water with concentrations adjusted to yield an absorbance value lower than 0.1. Slit widths for both excitation and emission were fixed at 4.0 nm. The quantum yields of developed GQD/300 obtained after 480 min of treatment were determined to be 0.069. Research works confirmed that although oxygen-containing functional groups present in GQDs make the material hydrophilic and offer sites for additional chemical functionalization, they also act as emissive traps resulting in reduced quantum yield.

### 3.4. Structural and Morphological Characterization of GQDs

**Figure 4:**
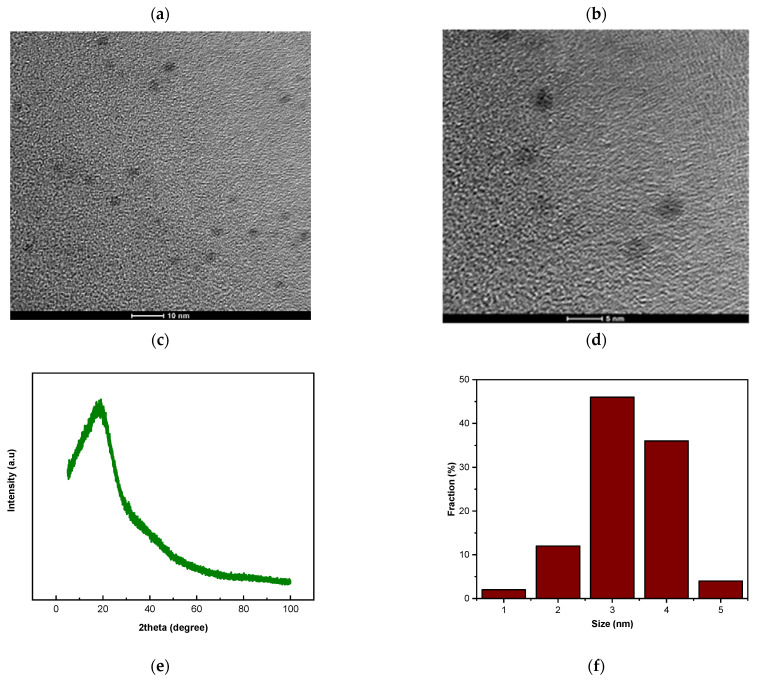

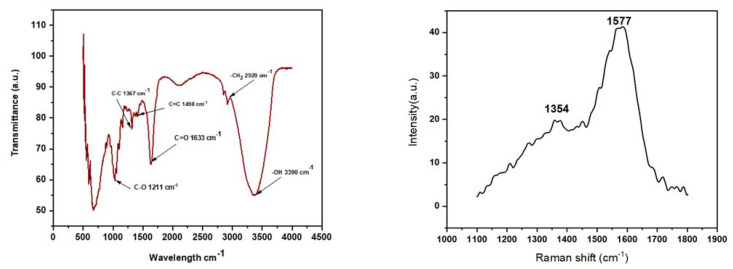
(**a**) TEM images of GQD/300 at 10 nm magnification (**b**) TEM images of GQD/300 at 5 nm magni-fication (**c**) particle size distribution of GQD/300 (**d**) XRD spectrum of GQD/300 (**e**) FTIR spectrum of GQD/300 (**f**) Raman spectrum of GQD/300.

The TEM images of the synthesized GQD/300 are shown in Figure 4a,b. The structure of GQD/300 examined by TEM confirmed that the GQD/300 were noted to have a size in the range of 2 to 5 nm. Figure 4c shows the particle size distribution of GQD/300. This is in line with the results obtained by a research work completed by Kumawat et al. [53]. The synthesis of the GQD/300 may be because of the carbonization of the solution during the heat treatment in the autoclave. The material’s carbonization degree will support controlling the size of the GQD/300 developed. The resultant GQDs are single dispersed sphere particles, as observed. The mean size of the GQD/300 particle is (Figure 4c) around 3 nm, with a comparatively tapered size distribution between 3–5 nm (based on statistic evaluation of over 100 quantum dots).

The XRD profiles in Figure 4d show that the GQD/300 have single broad diffraction peaks centered around 21°, which is attributed to the (002) lattice spacing of carbon-based materials with amorphous nature, a graphitic structure, suggesting that carbonizing eucalyptus powder produces graphitic structures. The peak is broad due to the small size of the GQD/300, and it is a typical band corresponding to highly disordered amorphous graphene quantum dots [35,36,78]. In several circumstances, the XRD patterns of GQDs are comparable to the bio-mass precursor peak with a minimal shift, as the main component of these materials is carbon, the only variation that matters are the peak intensity and broadness of the signal. The key difference between the precursor and resulting materials is peak sharpness and the XRD result shows the peak widening, which suggests the development of an amorphous nanoscale GQD structure.

The chemical bonding states of GQD/300 were investigated and characterized using FTIR spectra (Figure 4e). The FTIR spectra of GQD/300 exhibited strong absorption bands as seen in Figure 4e. The GQD/300 exhibited stretching vibrations of the carbonyl group –C=O at 1633 cm^−1^, hydroxyl group –OH at 3390.0 cm^−1^, and C=C stretching vibrations at 1498 cm^−1^ and –CH_2_ stretching at 2929 cm^−1^. Thus, it can be suggested that during hydrothermal polymerization, a carbonization reaction has occurred. The C–O bond stretching caused the peak to occur at 1211 cm^−1^. All of the findings matched with the results observed in earlier investigations [79,80,81].

GQD/300 Raman spectroscopy result is shown in Figure 4f and it revealed two distinct bands called D and G bands. The band D at 1354 cm^−1^ is linked to the crystalline characteristic of the compound and the vibrational characteristic of carbon atoms with dangling bonds. However, the band G at 1577 cm^−1^ is assigned to the crystalline nature of the compound as well as E2g vibration on photon mode of sp^2^ hybridization of carbon atom in the 2D hexagonal lattice of graphite structure(D,G) [82,83]. The ratio of the intensities of the disordered D band and the amorphous G band (D/G) is a typical approach to evaluate the homogeneity (degree of disorder or graphitization) of a quantum dot sample. The D/G ratio of the amorphous quantum-dots sample is greater. A reduced D/G ratio confirms a greater degree of graphitization in the specimen. In the current study, the D/G ratio of GQD/300 was noted to be 0.532 and this suggested that graphene quantum dots have a nano-crystalline graphite structure, which is nearly the same as the results formerly published [38,84,85].

### 3.5. Application of GQD/300 as PL Metal Ion Sensor

**Figure 5:**
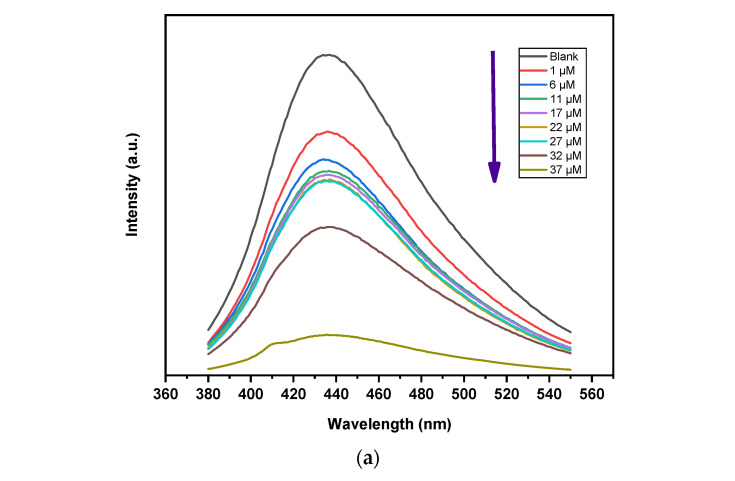

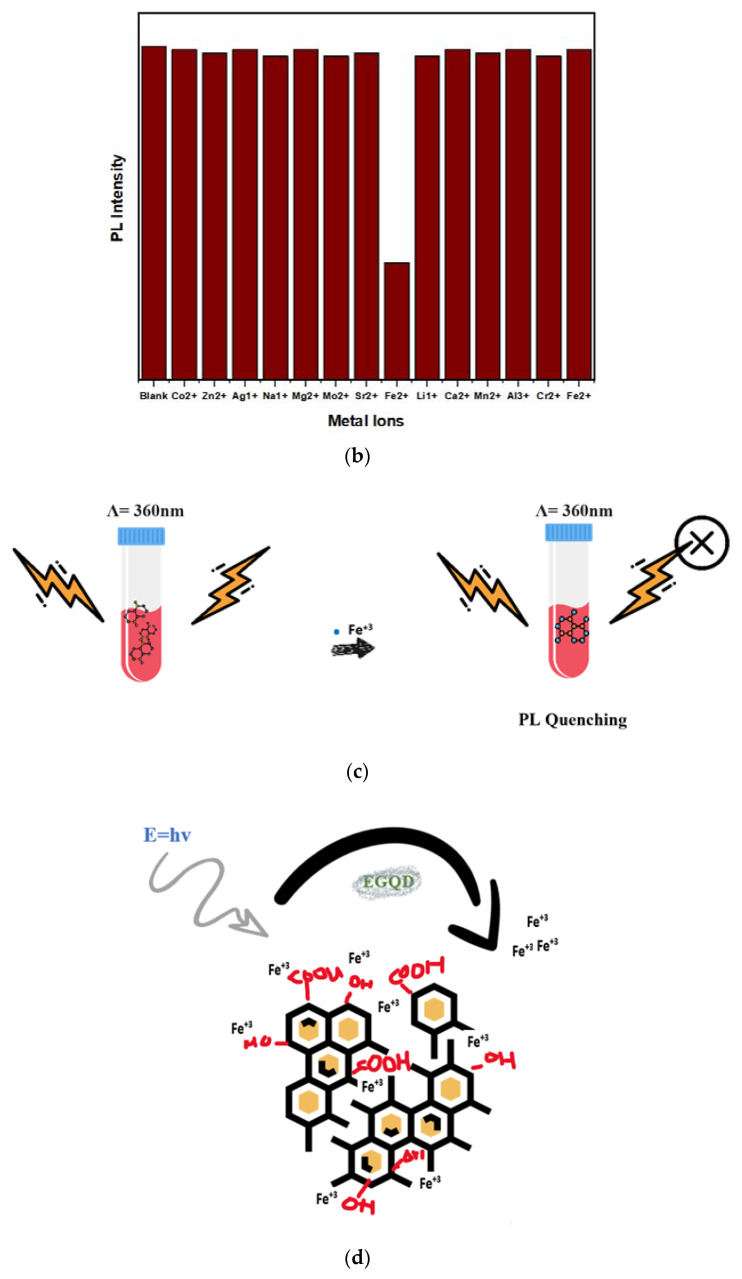
(**a**) PL spectra of GQD/300 in the presence of various concentrations of Fe^3+^ ions (**b**) comparison of the photoluminescence intensities of 55 g mL^−1^ GQD/300 solution containing various metal ions at an excitation wavelength of 360 nm. (**c**) Shows the schematic of fluorescence quenching. (**d**) Fluorescent quenching mechanism of the GQD/300 in presence of the Fe^3+^ metal ions.

By examining the variations in fluorescence intensity with different metal ion concentrations (1 μM, 6 μM, 11 μM, 17 μM, 23 μM, 28 μM, 32 μM, 37 μM, the variation in fluorescence intensity of GQD/300 was tracked. The variation in intensity was used to estimate the sensitive response of GQD/300. The analytical performance of the GQD/300 sensing system was performed in order to show the method’s sensitivity as well as for the quantitative study of the PL response of the GQD/300 towards different metal ions. The affinity (coordination) of several metal ions toward GQD/300 was assessed thoroughly under comparable experimental conditions. Figure 5b demonstrates that among sixteen different metal ions, the Fe^3+^ ions have the largest affinity (Figure 5b) for GQD/300, indicating the possibility of selective sensing by GQD/300. 

When illuminated, charge carriers in GQD/300 moved from the highest occupied molecular orbital to the lower unoccupied molecular orbital and then fluorescence appeared. Additionally, GQD/300’s primary dependence for their PL emission wavelength was particle size, which showed its effect on energy gap variation. GQDs that show smaller bandgap will be greater in size and vice versa [23,86]. For GQD/300 containing metal ion adsorbent, photoluminescence measurements were made at room temperature with an excitation wavelength of 360 nm. Interestingly, it was discovered that the maximal PL intensity of the GQD/300/Fe^3+^ combination significantly decreased upon increasing the metal ion concentration as reported by many authors. The generated histogram clearly demonstrated that the addition of metal ions caused PL alterations in GQD/300. With the inclusion of 1 µM of the metal ion Fe^3+^, a maximum 24% reduction in PL intensity for the GQD/300/Fe^3+^ combination is seen. The intensity of the PL peak was reduced by 37% by raising the Fe^3+^ quencher ion concentration from 1 µM to 6 µM. This demonstrated that the GQD/300 is extremely sensitive to Fe^3+^ even at very low quencher concentrations (1 µM). The GQD/300 displays significant PL emission and distinctive oxygenated surface functionalization in their as-synthesized state. Because of the presence of hydrophilic functional groups, the GQD/300 has good solubility in water. These GQDs are anticipated to be the best option for fluorescence sensing due to the existence of these functional groups as well as strong emissions. Several scientists used the quenching of carbon QDs by the addition of Fe^3+^ to accomplish this method. Fe^3+^ concentration and quenching % are correlated. In order to track how metal ions affect the intensity of the PL, a 360 nm wavelength was chosen. Different metal ions were added to the GQD/300 PL intensity to record them. According to Figure 5b, only Fe^3+^ ions out of a total of sixteen different metal ions significantly reduced the PL of GQDs when compared to the control (blank) sample. These findings imply that the GQD/300 can detect Fe^3+^ in a very selective manner. These findings can be explained as the functional groups located on the margins and base of GQDs’ increasing binding affinity. When ligands such as carboxyl and hydroxyl groups are present, they serve as a choice coordination site for the metal ions to diffuse and adhere to the GQD/300 and due to variations in charge transfer, changes in the local density of states, and changes in optical characteristics, this will result in a narrowing of the energy gap between GQD/300 and quencher. This results in static PL quenching without spectra shift which can be observed in Figure 5a. The significant affinity of Fe^3+^ ions toward the hydroxyl/carboxyl groups of GQD/300, which results in a stable complex, is connected to the mechanism of PL quenching. Figure 5c presents the fluorescent quenching mechanism of the GQD/300 in presence of the Fe^3+^ metal ions.

In general, the bare GQDs are very sensitive to several metal ions, and some transition or heavy metals could selectively quench fluorescence [87]. Other than the surface functionalized GQDs (that generate a specific combination of Fe^3+^), bare GQDs are also very selective for Fe^3+^ ions. Research works on the selective detection of Fe^3+^ have concentrated mainly on how Fe^3+^ quenches GQDs fluorescence. The unique coordination interaction between the phenolic hydroxy groups of the GQDs and Fe^3+^ ions or the energy/electron transfer process has been extensively used to describe the basis of fluorescence quenching [88]. Furthermore, few authors have explained this situation taking into account the pH values of GQDs solutions. Nevertheless, comprehensive justifications have not been suggested for why metal ions can be selectively detected employing the bare GQDs. Such a particular mechanism needs to be studied further. The majority of studies have recommended that the recognition mechanism arises from the specific coordination interaction between phenolic hydroxyl groups on the GQD surface and Fe^3+^ [89]. The subsequent electron-transfer process between the GQDs and Fe^3+^ brings fluorescence quenching dropping their fluorescence lifetime. Apart from inducing dynamic quenching, the GQDs could also develop complexes with Fe^3+^ for inducing the static quenching. Even though certain studies have employed this sensing mechanism, the idea that Fe^3+^ could be specially identified from other metal ions still lacks systematic research as well as experimental support [90].

During illumination, the metal ion was bonded to the GQD/300 surface by functional groups with electrostatic attraction or non-covalent bonds. Following the identification of particular metal ions (Fe^3+^), the distance between GQDs and Fe^3+^ ions was reduced, strengthening the GQD/300-Fe^3+^ interaction. This strongly promotes charge transfer between GQD/300 and Fe^3+^ ions, hence reducing GQD fluorescence. This quenching effect results from the inherent emission effect of excited state electron transfer by the absorption of photons between the metal ions and the fluorophore, which promotes electron–hole pair formation. The concentration of the target analyte affects the PL quenching of GQD/300 as well, allowing for the sensitive detection of deleterious metal ions. It has already been mentioned that until they are close to each other, fluorescence quenching of the fluorophore with the presence of metal ions is feasible in both the conjugated system and the unbonded state. A charge transfer-based sensing technique will have great sensitivity due to the high quenching efficiency of metal ions to the fluorescence of GQD/300. The probability of achieving a nonradiative transition increases with decreasing the distance between the lowest unoccupied (LUMO) and highest occupied (HOMO) molecular orbitals which results in a smaller emission gap [91]. Furthermore, because of structural relaxation during photoexcitation, the emission gap shrinks and overtakes the absorption gap. On the surface of the GQD/300, functional groups such as carboxyl, carbonyl, and hydroxyl may greatly promote the nonradiative transition through structural vibrations [92].

## 4. Conclusions

In the current study, we demonstrated a rapid, accessible, and inexpensively viable green preparation of GQDs using Eucalyptus tree leaves as the precursor. The current study reported on the synthesis of GQDs from the ethanolic extracts of eucalyptus tree leaves by a hydrothermal treatment technique at different heat treatment times and temperatures. This is a very sustainable way of preparing the GQDs, where the ethanol used will be recovered during the GQD preparation stage itself. The optical, morphological, and compositional analyses of the green-synthesized GQDs were carried out. It was observed that the product yield of GQDs showed the maximum yield at a reaction temperature of 300 °C. Furthermore, it was noted that at a treatment period of 480 min, the greatest product yield of about 44.34% was attained. The quantum yields of prepared GQD/300 obtained after 480 min of treatment at 300 °C were calculated to be 0.069. Moreover, the D/G ratio of GQD/300 was noted to be 0.532 and this suggested that the GQD/300 developed has a nano-crystalline graphite structure. The TEM images demonstrated the development of GQD/300 with sizes between 2.0 to 5.0 nm. Moreover, it was noted that the GQD/300 can detect Fe^3+^ in a very selective manner, and hence the developed GQDs were successfully used for the metal ion sensing application. The emerging field of GQD sensors for metal ion species should be studied more, with a perspective on the future of this extremely versatile material.

## Figures and Tables

**Table 1:** Product yield of GQDs at different reaction temperatures and constant heat treatment time.

Temperature (°C)	Treatment Time (min)	Product Yield (%)
240	120	10
260	120	13.24
300	120	22.33
320	120	19.25

**Table 2:** Product yield of GQDs at different treatment times and at a constant temperature of 300 °C.

Temperature (°C)	Treatment Time (min)	Product Yield (%)
300	360	22.33
300	400	27.00
300	440	35.88
300	480	44.34
300	520	39.67

## Data Availability

Not Applicable.
